# Pursuing Intracellular Pathogens with Hyaluronan. From a ‘Pro-Infection’ Polymer to a Biomaterial for ‘Trojan Horse’ Systems

**DOI:** 10.3390/molecules23040939

**Published:** 2018-04-18

**Authors:** Elita Montanari, Chiara Di Meo, Angela Oates, Tommasina Coviello, Pietro Matricardi

**Affiliations:** 1 Department of Drug Chemistry and Technologies, Sapienza University of Rome, P.le Aldo Moro 5, 00185 Rome, Italy; elita.montanari@uniroma1.it (E.M.); chiara.dimeo@uniroma1.it (C.D.M.); tommasina.coviello@uniroma1.it (T.C.); 2 School of Healthcare, Faculty of Medicine and Health, University of Leeds, Leeds LS2 9JT, UK; A.Oates1@leeds.ac.uk

**Keywords:** hyaluronan, intracellular infections, CD44, nano-carriers, antimicrobial delivery

## Abstract

Hyaluronan (HA) is among the most important bioactive polymers in mammals, playing a key role in a number of biological functions. In the last decades, it has been increasingly studied as a biomaterial for drug delivery systems, thanks to its physico-chemical features and ability to target and enter certain cells. The most important receptor of HA is ‘Cluster of Differentiation 44’ (CD44), a cell surface glycoprotein over-expressed by a number of cancers and heavily involved in HA endocytosis. Moreover, CD44 is highly expressed by keratinocytes, activated macrophages and fibroblasts, all of which can act as ‘reservoirs’ for intracellular pathogens. Interestingly, both CD44 and HA appear to play a key role for the invasion and persistence of such microorganisms within the cells. As such, HA is increasingly recognised as a potential target for nano-carriers development, to pursuit and target intracellular pathogens, acting as a ‘Trojan Horse’. This review describes the biological relationship between HA, CD44 and the entry and survival of a number of pathogens within the cells and the subsequent development of HA-based nano-carriers for enhancing the intracellular activity of antimicrobials.

## 1. Introduction

Intracellular pathogens are considered to be among the major bacterial public health threats [1]. The outcome of intracellular infections is largely due to the ability of pathogens to utilise specific cell receptors [2] and host components [3] for invading and subverting cellular activities. Evidences suggest a number of microorganisms can utilise hyaluronan (HA) and/or Cluster of Differentiation 44 (CD44) [4,5,6], an important receptor for HA [7,8,9], to promote attachment, invasion and replication within the cells. This ability has been recognised to be a potential factor in persistent infections and treatment failure.

Despite the ability of certain antibiotics to cross cell membrane, their intracellular efficacy can be poor due to: (I) intracellular concentrations below the minimum inhibitory value; (II) intracellular environment (e.g., acidic pH) that may affect the antibiotic activity; (III) antibiotic accumulation in subcellular compartments that are different from those in which pathogens reside.

**Figure 1:**
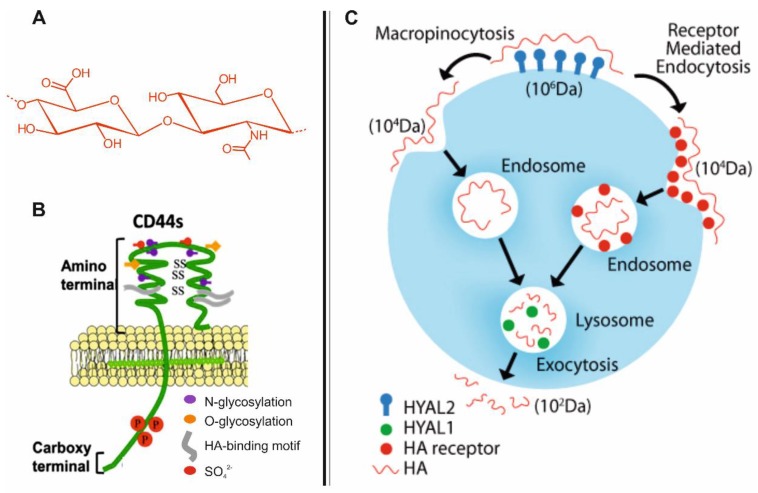
(**A**) Chemical structure of the HA repetitive unit. (**B**) Model structure of the standard CD44 receptor. (**C**) Schematic overview of HA endocytosis and degradation within the cells. ((**C**) reproduced with permission from © 2012 Racine R, Mummert ME. Published in Molecular Regulation of Endocytosis, IntechOpen, 2012, under CC BY 3.0 license. Available from: http://dx.doi.org/10.5772/45976 [19]).

In 1934, Karl Meyer and John Palmer isolated an unknown chemical substance from the vitreous body of bovine eyes, which contained two sugar molecules [10]. As one of these sugar molecules was an uronic acid, the name “hyaluronic acid” (HA) was coined by joining three words: *hyaloid* (vitreous) and *uronic acid.* Further work by Karl Meyer and his associates led to the resolution of the chemical structure of HA by the 1950s [11]. HA is a linear and non-sulfated glycosaminoglycan (Figure 1A), a poly-anionic polysaccharide composed of alternating d-glucuronic acid and *N*-acetyl-d-glucosamine monomeric units linked together through β-1,4 and β-1,3 glycosidic bonds. In early 1980s, Endre Balazs was successful in isolating a purified high molecular weight HA, which was used to produce plastic intraocular lenses for implantation [12]. In 1986, the term “hyaluronan” was introduced, to encompass the various forms the HA macromolecules could take: the acid form, hyaluronic acid, and its salts, such as sodium hyaluronate, which forms at physiological pH [13]. The injectable form of HA (‘Hyalgan^®^’) was approved in 1997, by the FDA for the treatment of pain associated with knee osteoarthritis by viscosupplementation [14] and, more recently, thanks to its biocompatibility, biodegradability [15] and its ability to provide high osmotic pressure and hydration, HA has found a market as a biomaterial in the cosmetic industry [14,16] and ophthalmology [17].

An important in vitro receptor of HA is CD44 [18] (Figure 1B); after the binding with HA, CD44 facilitates: (I) HA endocytosis [19] (Figure 1C) and (II) signalling events that generate a number of cell specific responses [18]. Cells which are known to highly express CD44 and internalise HA are keratinocytes [20], activated macrophages [21], fibroblasts [22], chondrocytes [23] and certain cancer cells [18,24]. The first three cell lines can act as ‘reservoirs’ of intracellular pathogens and, more interestingly, a number of works have shown these pathogens can utilise CD44 and/or HA for invading and surviving within such cells. Consequently, the incorporation/linkage of antimicrobials into HA-based nano-carriers represents a novel paradigm in the delivery of therapeutics against intracellular pathogens, as HA may enhance the sub-cellular targeting in addition to the efficacy of such antimicrobials by enabling the system to act as a ‘Trojan Horse’.

## 2. Biodistribution and Roles of HA

HA is a bioactive polysaccharide that naturally occurs in all living organisms [25], it is mostly found in the extracellular and pericellular matrices [26] such as the connective tissues, synovial fluid of joints and vitreous humour of the eye, however, intracellular locations such as the cytoplasm and vesicles, have also been documented [26]. In mammals, HA is synthesised by at least three synthases (HAS1, HAS2 and HAS3) [27] with expression of HAS genes appearing to be both tissue- and site-specific [28]. These enzymes (which are glycosyltransferases) differ from each other in their catalytic activities (HAS3 > HAS2 > HAS1) as well as in the size of their final products [29].

Degradation of HA typically occurs through a step-wise process [30] and its turnover can occur locally (in the cellular micro-environment) or at the tissue level. The local degradation includes: (I) HA binding, predominantly via CD44 [18] or via receptor for HA-mediated motility (RHAMM) [31,32]; (II) internalisation; (III) degradation within the cells by a series of coordinated enzymatic reactions in which HA fragments of decreasing size are progressively generated [33]. For the turnover at the tissue level, HA is released from tissue matrices, drained into vascular and lymphatic systems and then predominantly removed by liver and kidney [34]. The receptors involved in this pathway are HA receptors for endocytosis (HARE) [35] or lymphatic vessels endothelial HA receptors (LYVE-1) [36]. The enzymes required for HA synthesis and degradation are also involved in producing specific HA molecular weights, which in turn are related to specific functions of the HA fragments [37]. Furthermore, both location and HA concentration are also important variables in relation to the biological role that HA will take within the body [38]. High molecular weight HA (>1000–5000 repeating units) are typically extracellular, space-filling and have several structural functions which include lubrication of movable parts of the body, such as joints and muscles [39] and the maintenance of the viscoelasticity of connective tissues [39,40]. Moreover, high molecular weight HA controls the supramolecular assembly of proteoglycans in the extracellular matrix [38] and it is involved in the suppression of the angiogenesis [41] and immune-system [42]. In contrast, the small HA fragments appear to act as endogenous ‘danger signals’ [43], playing an active role in inflammation [44,45], immune-stimulation [46], cell detachment [47], migration [48] and tumour development [49].

Despite current data, there is a degree of uncertainty in HA biology; areas which require further exploration, include (I) the mechanism by which enzymes of synthesis and degradation of HA are able to cooperate for providing a proper HA size, (II) the binding of HA to CD44 and the subsequent internalisation within the cells; (III) the explicit role HA plays during the inflammation: evidences suggest in the alveolar tracts, released fragments of HA play a pivotal role in the host defenses, stimulating the innate immune responses, by activating TLR2 and TLR4 receptors promoting lung inflammation [50] and HA role in the resolution of inflammation, [51] and (IV) the role HA plays during the infection processes. The latter is one of the main focus of this review.

## 3. CD44-Mediated Uptake of HA in Host Cells

CD44 is a widely expressed family of class I transmembrane glycoproteins present on the surface of most mammalian cells [18,52]. CD44 is formed by an amino-terminal domain, which is known as the ‘link domain’ that enables the receptor to bind to HA as well as other glycosaminoglycans [53] (Figure 1B). The amino-terminal domain is separated from the plasma membrane by a short stem structure, which is followed by the transmembrane region and the cytoplasmic-tail [54,55]. After the binding with HA, CD44 works especially for two purposes: (I) to allow the HA endocytosis; (II) to trigger signalling events that induce a number of cell specific responses. In 2003, R. Stern proposed a general mechanism for the endocytosis of high molecular weight HA (hMWHA) and its catabolism within the cells [33] (Figure 1C). He proposed that hMWHA (M*_w_* ≥ 1 × 10^6^) is first degraded by the combined action of HA receptors and hyaluronidase2 (HA_ase_2) into intermediate-sized fragments (M*_w_* ≈ 1 × 10^4^) and then it is taken up by the cells. These fragments are then delivered to endosomal and lysosomal vesicles where a further catabolism could occur by HA_ase_1, coordinated with the activity of two specific lysosomal enzymes; finally, HA fragments are exocytosed [33].

HA binding and uptake through CD44 appear to be two separate events that often do not take place simultaneously. Evidences suggests that the HA uptake requires the acylation of the CD44 cytoplasmic tail [56] which can be a cell type specific event. Specifically, it was reported CD44 is associated with cholesterol-rich lipid rafts [57] and this association is dependent on the palmitoylation of both Cys286 and Cys295, which are in the highly conserved transmembrane domain and in the proximal cytoplasmic domain of CD44, respectively [58]. The prevention of CD44 localisation within lipid rafts blocks HA internalisation as well as the turnover/cycling of the receptor itself, but does not interfere with the ability of the receptor to bind to HA [56]. This evidence may explain why HA is not internalised in all CD44 expressing cell types.

Overall, cells that highly express CD44 and take up HA, leaving aside cancer cells [18,24], are keratinocytes [20], activated macrophages [21], fibroblasts [22] and chondrocytes [23]. In 2001, Tammi and colleagues showed keratinocytes express high level of CD44 and are able to internalise exogenous HA; however, HA and especially HA oligosaccharides can also enter keratinocytes via non-receptor mediated pathway [20]. Intracellularly, HA can be found in small vesicles with a diameter of ~100 nm, which are close to the plasma membrane and in larger perinuclear structures (>1 µm). Interestingly, a similar HA intracellular profile was found in liver endothelial cells [59]. These cells showed a greater ability to internalise HA, particularly into vacuoles with a diameter ranging from 0.3 to 1.2 µm, with the majority located close to the perinuclear region. The HA binding and internalisation profile has also studied in healthy human skin, normal scar and hypertrophic scar fibroblastic cell lines. These cells lines showed similar binding as well as internalisation curves of HA for all cells tested [24]. Moreover, normal scar fibroblasts showed greater ability to generate HA-derived partial degradation products.

Alveolar macrophages reside in the respiratory tract and alveolar space, where they are responsible for the uptake and clearance of pathogens as well as debris. These cells bind and take up HA in a CD44-dependent manner [60]; once internalised HA was found in the cytoplasm. Evidences also suggest that these cell types are the only immune cells that show to bind high levels of HA under homeostatic, non-infectious or non-inflammatory conditions, in both rodents and humans [58].

## 4. Role of CD44 and HA in the Uptake and Proliferation of Intracellular Pathogens

Keratinocytes, macrophages and fibroblasts can all act as ‘reservoirs’ for intracellular pathogens. Interestingly both HA and CD44, are utilised by a number of microorganisms to facilitate their invasion of such cells and persistence within the cellular micro-environment (Table 1). For example, *Streptococcus pyogenes* has been shown to be able to attach to epithelial cells through its HA-rich polysaccharide capsules, which mediates attachment to CD44 receptors on pharyngeal and epidermal keratinocytes [4,61] facilitating colonisation and infection in the throat and skin [61]. HA also appears to play a key role in the adherence of *Mycobacterium tuberculosis* to human lung epithelial cells (A549) [5]. Evidence suggest that *M. tuberculosis* utilises extracellular DNA-binding proteins to attach host cells through HA, indicating that HA represent the major binding site of *M. tuberculosis* in A549 cells; whilst CD44 appears to be involved in the binding and subsequent cellular internalisation of *M. tuberculosis* in murine primary macrophages [7]. CD44 is also implicated in the cellular uptake of *Staphylococcus aureus* in human neutrophils [8], possibly influencing the pathogen phagocytosis through its structural and functional linkage to the cytoskeletal microfilaments. A similar outcome was obtained for the cell internalisation of *Shigella* spp. in epithelial cells [62] where it appears that CD44 associates with *Shigella* spp. through IpaB, a protein which is secreted by the pathogen upon cell contact [62]. This IpaB-CD44 interaction led to the transduction of signals which participate in the cytoskeletal rearrangements and the subsequent internalisation of the pathogen within the cells. CD44 has also been shown to facilitate the intracellular growth of *Listeria* spp. in murine primary macrophages and fibroblasts. However, this may not be an ubiquitous effect; as comparisons, in *Salmonella enterica* serovar Typhimorium, CD44 did not play a role in their intracellular growth [9].

Once internalised, the ability of the microorganism to proliferate intracellularly is another advantage to survival. HA also seems play a role in this stage of infection. Evidence suggests that HA is important for the growth of the parasite *Leishmania* in primary and RAW 264.7 macrophages [6]. A study by Naderer et al. [6] indicates that HA is taken up by infected macrophages and is transported to the phagolysosome where *Leishmania* replicates; once internalised, HA provides *Leishmania* with essential nutrients for growth and virulence. With the aim to investigate this aspect the strategy adopted by the Nader group was generate *N*-acetylglucosamine (GlcNAc) acetyltransferase (GNAT) deficient *Leishmania* (Δgnat). This mutant was unable to grow or survive even when macrophages were cultivated in the presence of exogenous GlcNAc, suggesting intracellular HA provides *Leishmania* with essential carbon sources [6].

Other microorganisms have also been shown to utilise HA as a nutrient source for intracellular groth; for example strains of *M. tuberculosis* and *Mycobacterium bovis bacillus Calmette-Guerin* (BCG, an attenuated strain of *M. bovis* and a live vaccine against tuberculosis) have been shown to be able to utilise HA as a carbon source for proliferation [63]. In order to investigate this, ^3^H-labeled HA was added to an infection cell culture model where it was found to be incorporated into the live BCG, demonstrating HA uptake by the pathogen. Further work using l-Ascorbic acid 6-hexadecanoate (Vcpal), which is an inhibitor of HA_ase_, suppressed the enhancing effect of HA on the growth of *Mycobacteriae*, suggesting: (I) short HA chains are preferred as a carbon source; (II) *Mycobacteriae* utilise the exogenous HA [63].

Further experiments by the Matusmoto group also demonstrated that both BCG and *M. tuberculosis* grew when co-cultured with HA-synthase1 (HAS1) and HAS3 (which synthesise HA with a broad range of molecular weights, ranging from 2 × 10^5^ to 2 × 10^6^) but not HAS2 (which synthesises HA with molecular weights higher than 2 × 10^6^), confirming shorter HA chains are preferential for growth. Specifically, HAS1 appeared to be the major HA synthase in *Mycobacteriae*-infected mouse lungs [63] Treatment with hyaluronidase inhibitors (such as Vcpal, apigenin or quercetin [64]) could be an interesting approach to begin to give both an indication about which size range is preferential for growth and information about the intracellular or extracellular use of HA by the pathogen. However, to confirm the use of intracellular HA, radiolabelled HA should be also employed. Another interesting approach could be the use of HA synthesis inhibitors (such as 4-methylumbelliferone, 4-MU) [65]. Among the inhibition mechanisms, 4-MU appears to work as a competitive substrate for UDP-glucuronosyltransferase (UGT), which is an enzyme involved in HA synthesis [66]. The application of such treatment should confirm the need of certain pathogens to use HA for growth and virulence. Interestingly, the utilisation of HA_ase_ inhibitors, such as Vcpal [67], apigenin or quercetin [64] have been shown to suppress the growth of *Mycobacteriae* in mouse lungs, evidencing that HA_ase_ or a potential transporter of short size HA fragments could be potential targets for therapies against such pathogens.

Taking together these data, it is reasonable to assume HA may be a suitable biomaterial for building nano-carriers to target intracellular pathogens, acting as ‘Trojan Horse’, as: (I) a number of host cells (e.g., keratinocytes and macrophages) highly express CD44 and internalise HA; (II) HA can enter cells through CD44 receptor that is also used by such pathogens for the cell invasion; (III) like other nanoparticles, HA nano-carriers can be engineered in order to target sub-cellular compartments (e.g., lysosome or cytoplasm) where the microorganism grows and replicates; IV) HA nano-carriers may be cleaved by HA_ase_ that are produced by several pathogens, such as *Staphylococcus* spp. and *Streptococcus* spp. [68], facilitating the release of the drug in situ. Moreover, the depolymerisation/degradation of HA can also occur in the presence of host enzymes, free radicals [15], and at low pH values, leading to the drug release intracellularly, thus guaranteeing the efficacy of the targeted therapy also against microorganisms that typically do not produce HA_ase_.

## 5. HA-Based Nano-Carriers in Drug Delivery

In recent years, HA has received enormous attention as a biomaterial for building nano-carriers, thanks to its biocompatibility, low-toxicity, biodegradability, hydrophilicity, ability to protect the entrapped drug and to enhance the solubility of hydrophobic molecules. Furthermore, HA chains can be easily functionalised, in order to develop materials suitable for drug delivery. Chemical modifications of HA have been extensively reviewed [69,70] and target three functional groups: the carboxylic acid group, the primary and secondary hydroxyl groups, and the *N*-acetyl group (following deamidation). In the last decades, several kinds of HA-based nano-carriers have been developed, including self-assembling nanohydrogels (NHs) [71,72,73], covalently [74,75] or physically cross-linked nanoparticles, HA-coated liposomes [76,77] or inorganic nanoparticles [78,79] and bio-conjugates [80,81], and employed for a wide range of applications [82,83]. A number of stimuli-responsive HA nano-carriers have been also developed for the targeted and responsive delivery of therapeutics [84,85].

For example, HA-based nanoparticles are produced by using a number of strategies and are classified by the type of cross-linking from which they are formed: the most common nanoparticles are made by hydrophobic associations [71], chemical cross-linking [74] or electrostatic interactions [86]. HA nanoparticles made up of hydrophobic associations are usually obtained through the partial hydrophobisation of HA; both hydrophobic molecules and/or hydrophobic long chains can be grafted to HA, to obtain self-assembled nano-structures with internal hydrophobic domains, in aqueous environment. Specifically, 5β-cholanic acid [73], cholesterol [71], 2′3′4′5′-tetrabutyrilriboflavin [72], PLGA [87] and PEG-PCL [88] have been successfully linked to HA, allowing the formation of HA nanoparticles, usually named nanohydrogels (NHs). Self-assembling HA NHs can be used to deliver a wide range of therapeutic molecules or polypeptides [71,89]. However, the HA poly-anionic nature represents a limitation in encapsulating negatively charged macromolecules such as siRNA and DNA. To overcome this drawback, HA chains have been modified with mono-functional fatty amines (with different alkyl chain lengths) or cationic polyamines, such as polyethyleneimine, or poly(l-lysine), in order to achieve self-assembled HA NHs, capable to physically encapsulate and deliver genetic material [90].

Chemically cross-linked nanoparticles are usually more stable than the physically cross-linked analogues. However, the methods used for forming cross-linked nanoparticles, such as the micro-emulsion method [91], generally require high energy sources and drastic conditions (such as high speed mechanical stirring or the use of organic solvents and surfactants), which, for example, represent a limitation for the entrapment of sensitive molecules. Moreover, the permanent cross-linkages may inhibit drug release at the target site, resulting in a reduced therapeutic efficacy. Therefore, several degradable linkages including di-sulfide [92] and other pH-sensitive derivatives [93], have been used to obtain chemically cross-linked stimuli-responsive nanoparticles. For example, HA-boronate derivative has been synthesised with the aim to develop pH-responsive and chemically cross-linked nanoparticles [85], by spontaneously forming boronate esters with polycatechols, by very mild conditions. The same strategy was also applied for the development of pH-responsive HA bio-conjugates with a number of diol/catechol-based therapeutics [81].

The ionotropic gelation process represents another useful technique to prepare physically cross-linked HA nano-carriers. An example is represented by HA/chitosan nanoparticles [86]: the strong ionic interactions between the positively charged chitosan amino groups and the negatively charged HA carboxylic groups allow the nanoparticle network’s formation.

Among the advantages that HA-based nano-carriers offer, the binding of HA to CD44, is one of the most relevant; this property ensures both an active targeting to cells that over-express CD44 and the intracellular delivery of therapeutics. Among the cells, certain cancers, such as breast cancer, over-express CD44 and, for this reason, HA-based nano-carriers have been especially studied for cancer therapy and theranostics purposes. The synthesis and application of HA-based nano-carriers for targeting tumours have been extensively reviewed in a number of works [82,94], and therefore will be omitted here. However, it should be pointed out, HA is not internalised in all CD44 expressing cell types: therefore, tumours that highly express CD44 may take up only a little amount of HA [95].

The ability of HA to cross the cell membrane is another important advantage; this attribute represents an extremely useful strategy to deliver certain drugs intracellularly. Small molecules [87,88], poly-peptides [65,89,96] and genetic material [86,97] have been efficiently loaded into HA-based nano-carriers and delivered intracellularly. For example, HA naturally interacts with several proteins inside the body (hyaladherins) [38] (a number of biological functions of HA are attributed to this specific binding). Therefore, HA-based nano-carriers could represent a useful system to conjugate peptides [96] or proteins [71] and deliver them within the body, preserving their activity and increasing their stability and availability within the tissues. However, the shortcomings of using HA are its rapid clearance from the blood circulation, due to its recognition by HA receptors of reticuloendothelial system organs, such as the liver and spleen and subsequent degradation. This is the reason why, PEGylation is typically used for reducing HA degradation and prolongs its circulation within the body [98]. However, PEGylation could significantly affect the HA binding affinity to CD44 receptors, decreasing the nano-carrier cellular uptake in the desired site of action. Therefore, the degree of functionalisation of HA with PEG and the PEG molecular weight are fundamental parameters that should be controlled, to increase the nano-carrier circulation in the blood and to decrease their liver uptake, without affect their internalisation in the targeted site.

**Figure 2:**
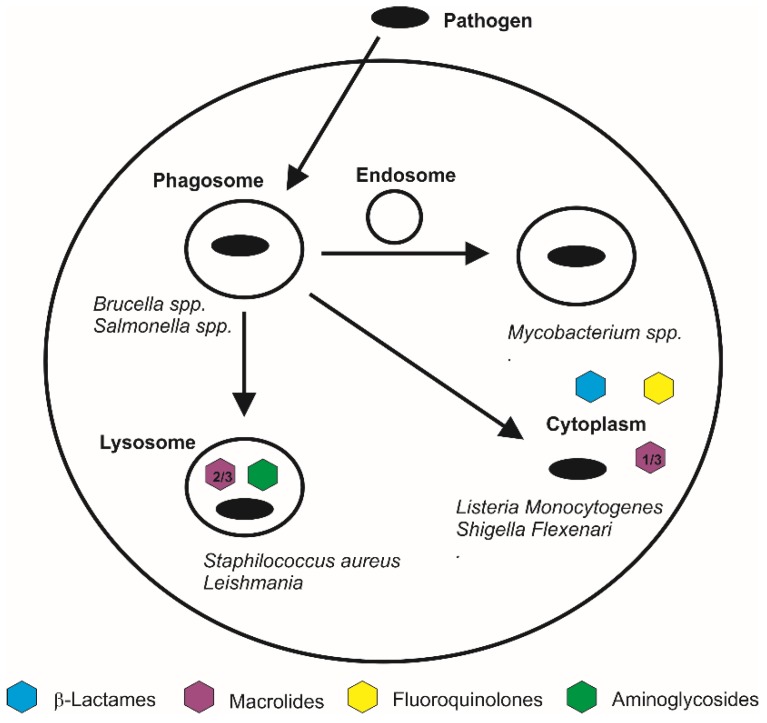
Scheme of the intracellular fate of several pathogens and antibiotics.

Keratinocytes, activated macrophages and fibroblasts are cells that express CD44 and highly take up extracellular HA, representing another interesting target for HA-based nano-carriers. Recently, the cellular uptake of HA/chitosan nanoparticles by activated macrophages, for a targeted therapy and CD44-mediated nucleic acid delivery, has been investigated in depth [97,99]. All these cell types can act as ‘reservoirs’ for facultative or obligate intracellular pathogens, therefore HA-based nano-carriers may represent an interesting approach for enhancing the targeting and intracellular uptake of antimicrobials, opening novel opportunities in this field. In this respect, it should be noted that the sub-cellular accumulation of antimicrobials in the same site of infection (e.g., endosome, lysosome, cytosol) may represent a key point for the effectiveness of the treatments, as different microorganisms can invade and replicate in different sub-cellular compartments (Figure 2). For example, *Mycobacteriae* can survive and replicate within macrophages by resisting lysosomal delivery by residing in early phase endosomal compartments [100], whilst microorganisms such as *Salmonellae* [101] and *Brucellae* [102] survive by preventing vacuole-lysosome fusion and pathogens such as *Shigella* spp. [103], *Listeria* spp. [104] and *Rickettsiae* [105] are able to escape from phagosomes and survive in the cytoplasm. To survive and disseminate intracellularly *S. aureus* [106,107] and Leishmania [6] appear to resist the fusion of phagosomes with lysosomes allowing them to multiply within the phagolysosomes of macrophages. Consequently, when treating an intracellular infection, a suitable antibiotic should be chosen in order to ensure drug concentration: (I) is above the minimum inhibitory concentration and (II) is delivered to the site of infection. Depending on their physico-chemical properties, antibiotics accumulate in different cell compartments at various concentrations [108,109]; typically, weak bases tend to accumulate in membrane-bound acidic compartments, whereas weak acids are excluded from those sites. Specifically, aminoglycosides [110] and macrolides [111] predominantly accumulate in lysosomes, quinolones accumulate in the cytoplasm [111], whereas β-Lactams [112] have been shown to accumulate at low level within the cells (predominantly in the cytoplasm) likely due to their acidic character.

Therefore, among all the drug delivery systems for antimicrobial activity, the HA-based nano-carriers may represent a novel paradigm for targeting to cells where pathogens persist, enhancing the intracellular drug concentration in the specific sub-cellular compartments. Indeed, such nano-carriers can be customised in order to target lysosomes [113] or to escape the endosome, being released into the cytoplasm [114].

## 6. HA-Based Nano-Carriers for Targeting Sub-Cellular Compartments

### 6.1. Lysosomal HA-Based Nano-Carriers

Even if a number of works have shown HA-based nano-carriers, are internalised by cells especially by CD44 receptors [115,116,117], the subsequent intracellular trafficking of these nano-systems is often not clear or is not investigated. It has been demonstrated self-assembled HA-cholesterol nanohydrogels (HA-CH NHs) (HA M*_w_* = 2.2 × 10^5^, CH degree of functionalisation = 15% (mol/mol, %), mean diameter~180 nm) quickly accumulate (within 1 h) in acidic endosomal and lysosomal compartments of human keratinocytes (HaCaT cells), reaching the highest co-localisation with those vesicles in 4 h [113]. Intracellularly, ApoTome analysis showed HA-CH NHs located into vesicle-like structures, those with a diameter of approximately 0.3 µm close to the plasma membrane and those in larger vesicles, with diameter up to 1.5 µm close to the nucleus. A similar outcome was reported by Tammi and colleagues (2001) using free HA in rat epidermal keratinocytes [20].

Self-assembling oleic acid-ethylendiamine nanoparticles, coated with HA (mean diameter~150 nm) showed co-localisation with lysosomes of colon cancer cells (HCT-116) over 6 h. Moreover, it was found that this nano-system is taken up by cells through both CD44 and clathrin-dependent endocytosis routes [117].

The intracellular pathway of polycarbonate: polyethylene: cholesterol (65:5:30) liposomes grafted with HA has been investigated on human lung carcinoma epithelial cells (A549) and human breast cancer cell lines (MB-231 and MCF7) [118]. A549 and MB-231 represent CD44 positive cell lines, whilst MCF7 represents the CD44 negative one. Several HA M_w_ (from 5.0 × 10^3^ to 1.6 × 10^6^) and degree of grafting density (HA final amount ranging from 0.2 to 1.5 mg) have been employed with the aim to investigate the impact of these parameters on the liposome uptake (the mean diameter ranged from 120 to 180 nm). Results showed HA-liposomes bind to CD44; this binding increases by increasing either HA M_w_ or grafting density (this trend was evident in A549 or MDA-MB-231 cells, but not in MCF7). Moreover, CD44-mediated uptake of HA-liposomes happened through lipid raft-mediated endocytosis (that is a cholesterol-dependent route) and it was independent from clathrin-coated vesicles or the caveolae or macropinocytosis pathways. HA-liposomes were found to be predominantly localised in acidic endosomal and lysosomal compartments [118].

Taking together these results, it is reasonable to assume that negatively charged and amphiphilic HA-based nano-carriers (formed by hydrophobic core/domain/particle and HA shell, and showing a mean diameter smaller than 200 nm) may enter cells especially through CD44 and predominantly accumulate into lysosomes, by following the endosomal-lysosomal pathway. Consequently, these nano-systems may be particularly suitable for targeting to pathogens that accumulate and replicate into these vesicles, such as *S. aureus*, *Leishmania* and, possibly, *M. tuberculosis*.

### 6.2. Cytosolic HA-Based Nano-Carriers

**Figure 3:**
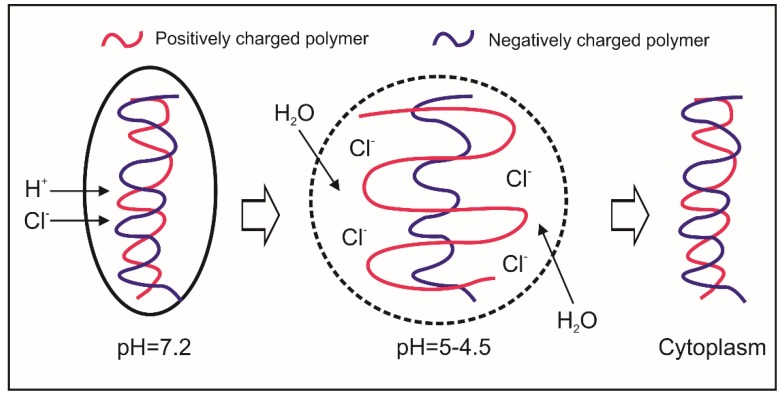
The ‘proton sponge’ hypothesis: H^+^ and Cl^−^ enter into the endosome, lead to osmotic swelling and finally to the endosome breakage.

The nanoparticle uptake through endosomal-lysosomal pathway may show some drawbacks: (I) in lysosomes, the low pH (~5) and the presence of an array of hydrolytic enzymes may lead to the destruction of such therapeutic molecules; (II) drugs do not reach the desired site of action, showing low effectiveness [119,120]. Therefore, recently, a number of strategies have been studied with the aim to develop nano-carriers that are able to escape the endosome or lysosome and as such accumulate into the cytoplasm [121]. In 1997, Jean-Paul Behr introduced the concept of ‘proton sponge’ effect [122] (Figure 3). After endocytosis, the buffering capacity of polycation/polyanion complexes will tend to both inhibit the action of the lysosomal nucleases (that have an acidic optimal pH) and alter the osmolarity of the vesicle [122]. The simultaneous occurrence of these two phenomena will firstly cause the swelling of endosomes/lysosomes and then the breakage of the vesicle membrane, leading to the release of ‘cargo’ into the cytoplasm. Nano-carriers able to exploit these properties are typically made of positively charged macromolecules such as polymers with low pKa amine group (e.g., polyethylenimine, poly-l-lysine, chitosan) or cationic lipids, which have been especially employed for gene therapy [123]. HA/chitosan nanoparticles represent a typical example of nano-carrier successfully designed for gene transfection (e.g., DNA, siRNAs) [124]. Their intracellular trafficking has been studied in phagocytic cells (e.g., macrophages) [114] and human epithelial cell lines derived from the conjunctiva and the cornea [124], showing the ability of HA/chitosan nanoparticles to escape lysosomes and target to the cytoplasm. Other examples of HA-based nano-carriers showing a strong ‘proton sponge’ effect and able to release the cargo in the cytoplasm, are represented by core/shell nanoparticles formed of poly(β-amino) ester coated with HA [125] and self-assembled HA-g-poly(l-histidine) micelles [126]. In order to enhance the endosomal/lysosomal breakage, a photochemically triggered self-assembling HA-based nanoparticle has been developed [127] by simultaneously linking a positively charged polymer poly-(diisopropylaminoethyl aspartamide) and a photosensitizer (chlorin e6) to the carboxyl and hydroxyl groups of acetylated HA, respectively. For example, these nano-carriers may be employed for targeting to microorganisms that typically replicate in the cytoplasm, such as *Shigella* spp. and *Listeria* spp.

The endosomal escape can be also achieved by using fusion proteins able to catalyse the membrane fusion between the particle and endosomes or to generate a pore on the membrane or the lysis of the membrane in order to empty their ‘cargo’ in the cytoplasm [128]. Also the use of cationic lipids represents another strategy that could be adopted for destabilizing the endosome membrane [129]. Indeed, after endocytosis, cationic lipids form ion pairs with the anionic ones, leading to the release of ‘cargo’ in the cytoplasm [130]. However, to the best of our knowledge, HA-based nano-carriers which exploit these properties have not been developed, yet.

## 7. The Application of HA-Based Nano-Carriers for the Intracellular Delivery of Antimicrobials

With increasing interest in the potential application of HA-based nano-carriers for the intracellular delivery of antimicrobials, there have been a growing number of investigations looking at their in vitro and in vivo efficacy. The use of HA-based nano-carriers for antimicrobial purposes could show several advantages in comparison to those of nano-carriers made of other polymers/materials: (I) a number of host cells (e.g., keratinocytes and macrophages) express CD44 and highly internalise HA, providing an active targeting; (II) HA can enter cells through CD44, the receptor also used by pathogens for the cell invasion; (III) HA-based nano-carriers may be cleaved by HA_ase_ that are produced by several pathogens, such as *Staphylococcus* spp. and *Streptococcus* spp. [68], thus facilitating the release of the drug in situ. The depolymerisation/degradation of HA can also occur in the presence of host enzymes, free radicals [15], and at low pH values, leading to the intracellular drug release, thus guaranteeing the efficacy of the targeted therapy also against microorganisms that typically do not produce HA_ase_. In this scenario, HA-based nano-carriers have been started to be developed and studied for the intracellular delivery of antimicrobials, both in vitro and in vivo.

A study by using bone-marrow derived macrophages infected with *M. tuberculosis* or *Mycobacterium avium* showed that treatment with an antimicrobial peptide (LLKKK18) entrapped into self-assembling HA-based NHs provided cellular/sub-cellular targeting and prevented the degradation of LLKKK18 by proteases [96]. Specifically: (I) the cytotoxicity of entrapped LLKKK18 was reduced in vitro; (II) the infected macrophages successfully internalised self-assembled HA NHs; (III) the targeting to *mycobacteriae* was enhanced using HA NHs even though the exact kind of vesicles in which the co-localisation occurred was not identified; (IV) experiments carried out with LLKKK18-loaded NHs showed the new system was more effective against both *M. avium* and *M. tuberculosis* than the free LLKKK18 in vitro and in vivo; (V) un-loaded HA NHs reduced the infection in mice.

It has also been reported that HA-streptomycin bio-conjugate showed the ability to enhance the antimicrobial activity of free streptomycin against *S. aureus* or *Listeria monocytogenes* in phagocytic cells (RAW macrophages) or non-phagocytic cells (VERO) [131]. Authors reported that HA did not improve the MIC of streptomycin on planktonic pathogens, but did show a high capability to enhance the antimicrobial activity against the two pathogens within phagocytic and non-phagocytic cells, in vitro and in vivo. Moreover, HA was able to enhance the streptomycin uptake, which was CD44-mediated and to reduce the ototoxicity and nephrotoxicity of streptomycin in mice. In previous study by the same research group, the capability of chitosan-based carriers to improve streptomycin activity intracellularly was demonstrated [132]. However, the cationic polysaccharide exhibited significant cytotoxicity at concentration higher than 500 µg/mL.

Investigations have also begun to focus on microorganisms that have not traditionally been thought as intracellular bacteria as there in increasing evidence suggesting that this mode of growth may facilitate persistent and chronic infections, and may be a cause of treatment failures. For example, axenic *P. aeruginosa* and *S. aureus* infected HeLa cells (a model cell line) were incubated with levofloxacin (LVF), a broad spectrum and highly active and cytosolic antibiotic [133], loaded into self-assembled HA-CH NHs. The reported results showed entrapped LVF was able to eradicate both intracellular microorganisms after only 2 h of incubation, whilst free LVF was ineffective intracellularly. A similar study was conducted on human keratinocytes (HaCaT cells) infected with *S. aureus* [113]; infected cells were incubated with LVF or gentamicin (GM)-loaded HA-CH NHs or their controls (free LVF, GM, NHs). These two antibiotics were selected as they have different intracellular pathways: LVF is a cytosolic drug, whilst GM is a lysosomal one. Results showed that NHs highly enhanced the antimicrobial activity of LVF against the intracellular *S. aureus*, but they did not improve the antibacterial activity of GM, which showed a significant effect without the employment of NHs. As it has been demonstrated that NHs co-localise with lysosomes of HaCaT cells and it is known that free LVF predominantly accumulates in the cytoplasm, these results suggested NHs may be able to change the intracellular fate of LVF from cytoplasm to lysosome, thereby targeting intracellular *S. aureus*, illustrating the importance of a targeted antibiotic treatment, and the the opportunity to enhance the intracellular activity of such antibiotics by using HA-based nano-carriers. It should be noted that, in both works, extracellular pathogens were previously eliminated, and the LVF-loaded NHs were tested only against the intracellular microorganisms. Though it is still not clear where the sub-cellular sites in which the loaded antibiotic was acting, the experiment was designed to remove any extracellular pathogen, so only intracellular microorganisms were counted after treatment. Moreover, as the free NHs was not effective against both intracellular and extracellular pathogens, it can be stated the antibacterial activity was only due to the intracellular LVF [113,133].

Recently, HA-amikacin was synthesised by ‘click’ reaction between HA-propargyl amide and amikacin-azide [134]. The obtained bio-conjugate was tested against planktonic *P. aeruginosa*, *S. aureus* and *L. monocytogenes*; HA did not improve the MIC of amikacin on planktonic pathogens, but did show a high capability to enhance the antimicrobial activity against the three pathogens within RAW 264.7 macrophages. The bio-conjugate was then tested in vivo, on mice infected intraperitoneally with *L. monocytogenes*, which received subcutaneous injection of HA-amikacin or its controls. Like the in vitro results suggested, the bio-conjugate showed an improvement of the antimicrobial activity of amikacin, evidencing the HA capability to enhance the efficacy of the antimicrobial against such intracellular pathogen. To the best of our knowledge, these are the only examples which describe the development and use of HA-based nano-carriers for targeting to intracellular pathogens. None of these works has shown a significant increase in toxicity of the loaded antimicrobials, both in vitro and in vivo. In contrast, a significant toxicity has been noted for chitosan-based nanoparticles [132], However, further studies are necessary to increase the understanding of the possible side effects that loaded antibiotics may cause (e.g., long-term side effects), the way in which the loaded antibiotics are acting against both intracellular and extracellular pathogens and the intracellular pathways that are involved in the uptake of these nano-formulations. Further investigations promise to be very productive.

## 8. Conclusions and Perspectives

The relationship between HA, CD44 and the invasion and survival of a number of pathogens within the cells is becoming increasingly evident. Several microorganisms utilise CD44 and/or HA to enter and replicate within the cellular micro-environment. Furthermore, a number of host cells highly express CD44 and internalise HA, ensuring an intracellular uptake of antimicrobials loaded into HA-based nano-carriers. This scenario makes HA a possible candidate for the development of ‘Trojan Horse’ systems to target intracellular microorganisms, thus overcoming the ineffectiveness of many antibiotics intracellularly. Previous works showed the ability of HA-based nano-carriers to enhance the intracellular activity of certain antimicrobials; however, little has been carried out in biology, microbiology and drug delivery fields, therefore further studies are necessary. HA is already extensively used for topical administration in cosmetics or ophthalmology. The incorporation of antimicrobials within HA-based nanogels may represent an interesting approach for enhancing the intracellular delivery of some drugs, thus targeting to pathogens that are a common cause of chronic topical infections (e.g., *S. aureus*) [113]. Moreover, the local HA administration could reduce the drawbacks due to the in vivo *u*ptake and catabolism of HA into cells that express LYVE-1 or HARE (e.g., liver and kidney). This aspect reduces the efficacy of the targeted therapy, representing an important disadvantage for HA-based injectable formulations. Several strategies have however been found for overcoming these drawbacks; for example, stealth HA nanoparticles have been shown to reduce the uptake by liver and kidney, improving the targeting to the desired site of action [98]. Toxicity is another aspect that must be taken into account: HA carriers should be able to enhance the intracellular uptake of antimicrobials and possibly, change their intracellular trafficking. So far, none of the authors reported a significant increase in toxicity of the loaded antibiotics in their experiments, both in vitro and in vivo but further work is necessary before clinical applications are fully realised. Furthermore, in the alveolar tracts, released fragments of HA play a pivotal role in the host defences stimulating the innate immune responses, by activating TLR2 and TLR4 receptors, thus inducing lung inflammation. There is a great deal of published and ongoing work into the role of HA during the inflammation processes even though a number of mechanisms are not yet clarified and with the indication that some pathogens may take advantage of these pathways for invading and surviving within host cells; this information is far from being clearly elucidated and our understanding in this area should be improved.

Antibiotic resistance is one of the major public health threats, and the novel approach based on HA nano-carries may represent an interesting strategy for overcoming some antibiotic failures in the treatment of intracellular infections.

## Figures and Tables

**Table 1:** CD44 and HA involvement in the host cell infections.

Pathogen	Cell Line	CD44 Role	HA Role
*S. pyogenes* [4]	Human keratinocytes	CD44 represents the main receptor for cell attachment.	HA-based capsules are synthesised for promoting the cell invasion.
*S. pyogenes* [61]	Murine epithelial keratinocytes	CD44 is found to be widely expressed in the site of infection, acting as a major cellular receptor for the cellular entry.	HA-based capsules are synthesised for promoting the cell invasion.
*M. tuberculosis* [5]	Human lung epithelial cells		Employment of extracellular DNA-binding proteins to attach host cells through HA.
*M. tuberculosis* [7]	Murine macrophages	CD44 involvement in the binding and subsequent cellular internalisation.	
*S. aureus* [8]	Human neutrophils	CD44 influences the pathogen phagocytosis through its structural and functional linkage to the cytoskeletal microfilaments.	
*Shigella* spp. [62]	Human epithelial cells	The IpaB-CD44 interaction leads to the transduction of signals that participate in the cytoskeletal rearrangements and the subsequent internalisation of the pathogen within the cells.	
*Listeria* spp. [9]	Murine macrophages and fibroblasts	CD44 facilitates the intracellular growth of the pathogen intracellularly.	
*M. tuberculosis* [63]	Human lung epithelial cells		Short HA chains are utilised as a carbon source for proliferation.
*M. bovis bacillus Calmette-Guerin* [63]	Human lung epithelial cells		Short HA chains are utilised as a carbon source for proliferation.
Leishmania [6]	Murine macrophages		HA acts as endogenous essential nutrient for the growth and virulence.
